# Peripheral B cells from patients with hepatitis C virus-associated lymphoma exhibit clonal expansion and an anergic-like transcriptional profile

**DOI:** 10.1016/j.isci.2022.105801

**Published:** 2022-12-15

**Authors:** Amanda N. Henning, Myagmarjav Budeebazar, Delgerbat Boldbaatar, Dahgwahdorj Yagaanbuyant, Davaadorj Duger, Khishigjargal Batsukh, Huizhi Zhou, Ryan Baumann, Robert D. Allison, Harvey J. Alter, Naranjargal Dashdorj, Valeria De Giorgi

**Affiliations:** 1 Department of Transfusion Medicine, Clinical Center, National Institutes of Health, Bethesda, MD 20892, USA; 2 Department of Gastroenterology, Mongolian National University of Medical Sciences, Ulaanbaatar 14210, Mongolia; 3 Liver Center, Ulaanbaatar 14230, Mongolia; 4 Center of Hematology and Bone Marrow Transplantation, First Central Hospital of Mongolia, Ulaanbaatar 14210, Mongolia; 5 Bloomberg School of Public Health, Johns Hopkins University, Baltimore, MD 21205, USA; 6 Onom Foundation, Ulaanbaatar 17011, Mongolia

**Keywords:** Epigenetics, Immunology, Virology

## Abstract

Chronic HCV infection remains a global health concern due to its involvement in hepatic and extrahepatic diseases, including B cell non-Hodgkin lymphoma (BNHL). Clinical and epidemiological evidence support a causal role for HCV in BNHL development, although mechanistic insight is lacking. We performed RNA-sequencing on peripheral B cells from patients with HCV alone, BNHL alone, and HCV-associated BNHL to identify unique and shared transcriptional profiles associated with transformation. In patients with HCV-associated BNHL, we observed the enrichment of an anergic-like gene signature and evidence of clonal expansion that was correlated with the expression of epigenetic regulatory genes. Our data support a role for viral-mediated clonal expansion of anergic-like B cells in HCV-associated BNHL development and suggest epigenetic dysregulation as a potential mechanism driving expansion. We propose epigenetic mechanisms may be involved in both HCV-associated lymphoma and regulation of B cell anergy, representing an attractive target for clinical interventions.

## Highlights

• Anergic-like gene signature seen in peripheral B cells from patients with BNHL/HCV
• Also observed expansion of lymphoproliferative and autoimmune-associated clonotypes
• Clonotype expansion correlated with epigenetic upregulation
• Epigenetic dysregulation may be involved in lymphoprogression and B cell anergy

## Graphical abstract

**Figure:**
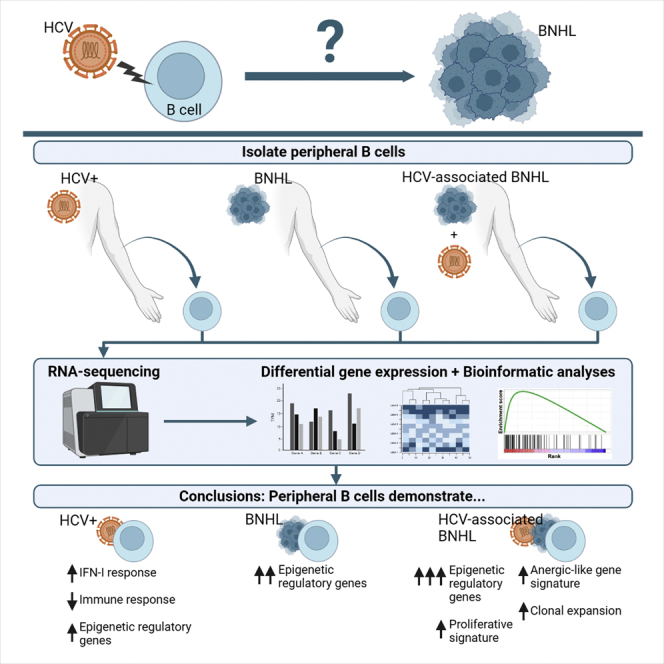

## Introduction

Globally, an estimated 71 million people are living with chronic hepatitis C virus (HCV) infection.^1^ Advances in direct-acting antivirals (DAA) have resulted in curative treatment options for chronically infected patients; however, global drug distribution and screening remain impediments to disease eradication, and new infections still outweigh cures.^2^ Chronic HCV infection plays a major role in the development of multiple hepatic and extrahepatic diseases. While its role in hepatic disease has been extensively characterized,^3^ ongoing work seeks to clarify the role of chronic HCV infection in extrahepatic diseases, especially for lymphoproliferative disorders (LPDs) such as mixed cryoglobulinemia (MC) and B cell non-Hodgkin lymphoma (BNHL). Multiple studies have identified a significant association between chronic HCV infection and BNHL development, with average relative risk ranging from 1.7 to 3.0, and elevated relative risks (>3) reported in areas with high HCV prevalence.^4^ HCV infection is more frequently observed in patients with marginal zone lymphoma (MZL) or diffuse large B cell lymphoma (DLBCL), although associated cases are also seen in other histologies.^4^ Clinically, successful treatment of HCV has been associated with improved overall survival in patients with MZL and DLBCL, and there have been reports of partial or complete lymphoma responses from DAA treatment alone.^5^

Although clinical and epidemiological evidence strongly support a causal role for HCV in the pathogenesis of BNHL, the biological mechanisms driving this association remain unclear. HCV-associated lymphomagenesis is thought to occur via two non-exclusive mechanisms: indirect transformation resulting from chronic BCR stimulation or engagement of CD81 by the HCV-E2 protein, or direct transformation resulting from HCV infection and replication within B cells.^4^^,^^6^^,^^7^ Evidence exists for both mechanisms. HCV RNA and indications of active viral replication have been observed in peripheral B cells from chronically infected patients, supporting direct transformation,^8^^,^^9^^,^^10^^,^^11^^,^^12^ and immunoglobulin (Ig) gene repertoires specific to chronic HCV infection have been observed in HCV-associated lymphomas, supporting the indirect transformation hypothesis.^6^

We performed transcriptional analysis on peripheral B cells from HCV-infected patients with or without BNHL, along with uninfected BNHL patients and healthy donor (HD) controls. In this way, we sought to detect shared and distinct transcriptional changes present in the periphery to identify potential mechanisms driving HCV-associated BNHL. We identified an anergic-like transcriptional signature and evidence of clonal expansion specific to peripheral B cells from patients with HCV-associated BNHL, and a pattern of epigenetic upregulation present in all patients with BNHL. Our data suggest epigenetic regulation may be involved in anergic signaling in HCV-associated BNHL and may represent a common mechanism driving lymphoprogression in viral- and non-viral-associated BNHL. These data present new areas of translational and clinical investigation to expand our understanding of both basic B cell biology and BNHL development.

## Results

### Transcriptional analysis of peripheral B cells from patients with B cell non-Hodgkin lymphoma with or without hepatitis C virus infection

Patients were recruited for this study from the First Central Hospital of Mongolia. Mongolia has one of the highest rates of HCV infection in the world,^13^^,^^14^ making it an ideal region for studying the relationship between HCV and BNHL. Whole blood samples were collected from patients with BNHL alone (BNHL; n = 6), chronic HCV infection alone (HCV; n = 9), HCV-associated BNHL (BNHL/HCV; n = 6), and healthy donors (HD; n = 6) (Table 1). The average age of the cohort was 52 (range 28-72) and 63% of patients were female. Quantification of HCV RNA revealed no significant difference between HCV-infected groups (Figure S1A), although 1 BNHL/HCV patient had undetectable levels and was considered a spontaneous responder. All samples were negative for HBV surface antigen, and BNHL and HD groups tested negative for anti-HCV antibodies. DLBCL was the predominant subtype within the HCV-associated BNHL cohort (n = 5/6), while the BNHL-only lymphoma group included cases of follicular lymphoma (FL; n = 1/6) and MALT lymphoma (n = 2/6). While these subtypes typically have an indolent disease course, two of these patients presented with stage III disease at the time of sample collection, indicating a more aggressive disease.^15^^,^^16^ In general, the majority of patients with lymphoma (67%, n = 8) presented with Stage III/IV disease (Table 1). Clinical testing indicated elevated levels of AST and ALT in patients with BNHL/HCV and HCV and no significant differences in albumin or platelet levels (Figures S1B–S1E). Calculated FIB-4 fibrosis scores demonstrated no significant differences across groups (Figure S1F), and the majority of our cohort (58%, n = 15) had scores consistent with an absence of advanced fibrosis, while only 4 patients (15%) had elevated scores, suggesting the presence of more advanced fibrosis.^17^

**Table 1:** Patient demographics and clinical characteristics

	Total (n = 27)	HD (n = 6)	BNHL (n = 6)	BNHL/HCV (n = 6)	HCV (n = 9)
Age					
mean ± SD	52.0 ± 12.8	47.7 ± 12.4	54.0 ± 9.6	55.8 ± 13.9	51.1 ± 15.3
Range	28 - 72	28 - 64	37 - 65	38 - 70	34 - 72
Female sex, n (%)	17 (63%)	2 (33%)	4 (67%)	5 (83%)	6 (67%)
HCV RNA (log_10_ IU/mL)					
mean ± SD	N/A	N/A	N/A	6.24 ± 0.69^a^	6.01 ± 0.57
Range	N/A	N/A	N/A	5.10 - 6.79	4.88 - 6.78
Clinical Features, mean ± SD					
FIB-4 Score	2.03 ± 2.42^b^	1.00 ± 0.38^b^	1.02 ± 0.5	4.34 ± 4.07	1.74 ± 1.49
AST, U/L	44.4 ± 40.9^b^	27.1 ± 15.8^b^	18.6 ± 4.7	83.5 ± 64.0	45.1 ± 26.1
ALT, U/L	45.4 ± 32.5^b^	35.7 ± 18.1^b^	18.0 ± 3.9	69.6 ± 48.4	53.0 ± 23.3
Albumin, g/L	41.7 ± 3.6^b^	43.8 ± 1.9^b^	40.4 ± 3.2	40.1 ± 4.9	42.5 ± 3.3
Platelets, x10^3^/μL	222.3 ± 63.3	224.8 ± 23.3	261.7 ± 44.3	167.3 ± 57.8	231.0 ± 76.6
B-NHL Subtype, n (%)					
DLBCL	N/A	N/A	2 (33.3%)	5 (83.3%)	N/A
FL	N/A	N/A	1^c^ (16.7%)	–	N/A
MALT lymphoma	N/A	N/A	2^c^ (33.3%)	–	N/A
MCL	N/A	N/A	–	1 (16.7%)	N/A
Subtype not determined	N/A	N/A	1 (16.7%)	–	N/A
Clinical stage, n (%)					
Stage I	N/A	N/A	1 (16.7%)	–	N/A
Stage II	N/A	N/A	1 (16.7%)	2 (33.3%)	N/A
Stage III	N/A	N/A	3 (50%)	4 (66.7%)	N/A
Stage IV	N/A	N/A	1 (16.7%)	–	N/A

ALT, alanine transaminase; AST, aspartate aminotransferase; DLBCL, diffuse large B cell lymphoma; FL, follicular lymphoma; HD, healthy donors; MALT, mucosa-associated lymphoid tissue; MCL, Mantle cell lymphoma.

a excludes 1 patient with undetectable RNA.

b missing value for n = 1 patient.

c FL case determined to be Stage III, cases of MALT lymphoma determined to be Stage II and III.

RNA-sequencing was performed on B cells isolated from PBMC to identify differentially expressed genes (DEGs; log2 fold change < -1 or >1, adjusted p value <0.05) between diagnosis groups. We observed the fewest DEGs (n = 127) between BNHL and HCV diagnosis groups and the highest number of DEGs (n = 1,267) between BNHL/HCV and HD. All other comparisons demonstrated an intermediate number of DEGs (Figures S1G and S1H, Table S1).

### Transcriptional signatures associated with chronic hepatitis C virus are altered in hepatitis C virus-associated B cell non-Hodgkin lymphoma

**Figure 1:**
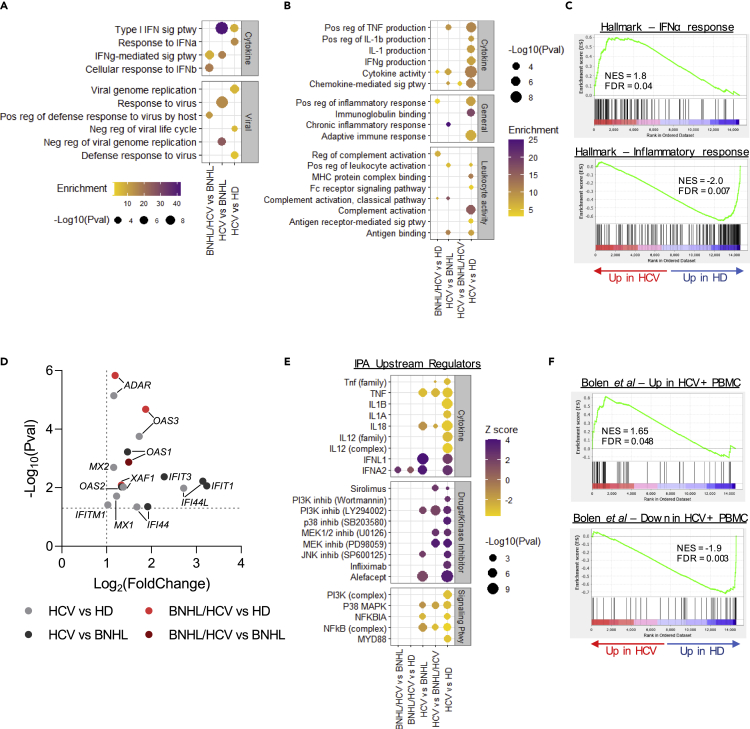
Transcriptional signatures associated with chronic HCV are altered in HCV-associated BNHL (A and B) Significant GO terms from upregulated (A) and downregulated (B) DEGs in HCV-infected groups related to cytokine activity, viral infection, leukocyte activity, and general immune response. GO terms are listed on the y axis. Circle size corresponds to GO term p value and color indicates GO term enrichment. (C) GSEA plots for expressed genes (ranked by fold change) in HCV vs HD comparison. (D) Select interferon-stimulated genes differentially expressed in HCV and BNHL/HCV comparison groups. Log2 fold change is listed on the x axis and –log10 (adjusted p value) is on the y axis. Dashed lines indicate DEG cutoffs. (E) Predicted upstream regulators of DEGs from Ingenuity Pathway Analysis on HCV and BNHL/HCV comparison groups. *Z* score color indicates whether molecules are predicted regulators of upregulated (positive *Z* score) or downregulated (negative *Z* score) genes, and circle size indicates p value. (F) GSEA plots of gene sets found to be up- and downregulated (top and bottom, respectively) in PBMC collected from patients with chronic HCV in a study by Bolen et al.^19^ Both plots show the enrichment of gene sets in expressed genes (ranked by fold change) in the HCV vs HD comparison group. FDR, false discovery rate; NES, normalized enrichment score. See also Figure S2, Tables S1 and S2.

In patients with chronic HCV infection, gene ontology (GO) analysis of differentially upregulated genes identified significantly enriched GO terms corresponding to Type I interferon (IFN-I) signaling and viral infection. This enrichment was observed in DEGs from HCV-only patients relative to HD and BNHL patients, but was not seen compared to dual diagnosis (BNHL/HCV) patients. Furthermore, IFN-I and viral-related GO term enrichment were less pronounced in samples from patients with BNHL/HCV relative to other groups (Figure 1A, Table S2). GO analysis of downregulated DEGs identified the enrichment of GO terms related to inflammatory cytokine production, the immune response, and leukocyte activity in HCV samples relative to other groups, but this enrichment was not as pronounced in BNHL/HCV samples (Figures 1B and S2A, Table S2). Similar trends were observed using gene set enrichment analysis (GSEA), where the Hallmark IFNα Response gene set was positively enriched in HCV-only samples but not in BNHL/HCV samples, and the Hallmark Inflammatory Response and TNFα Signaling gene sets were negatively enriched (Figure 1C and Table 2, Figure S2B). At the gene level, IFN-I and viral-related GO and GSEA enrichment were driven by the upregulation of a number of interferon-stimulated genes (ISGs).^18^ Specifically, we observed significant increases in the expression of members of the IFIT and IFITM families (*IFIT1/3*, *IFITM1/3*), oligoadenylate synthases (*OAS1/2/3*), and myxoma resistance protein 1/2 (*MX1/2*), among others (Figure 1D). An investigation of downregulated genes identified a broad assortment of genes with roles in the inflammatory pathway, including many pro-inflammatory cytokines and chemokines (*IL1B, IL32, CCL3/4/5, CXCL8*), Fc and Fc-like receptors (*FCAR, FCER1A/1G, FCGR3A, FCRL6*), and genes involved in BCR (*ITK*) and NFκβ (*S100A8, TLR4, NLRP3*) signaling (Figure S2C).

**Table 2:** Gene set enrichment analysis

		BNHL/HCV vs HD		BNHL/HCV vs HCV		BNHL/HCV vs BNHL		BNHL vs HD		HCV vs BNHL		HCV vs HD	
**Type I IFN gene sets**	# Genes	NES	FDR	NES	FDR	NES	FDR	NES	FDR	NES	FDR	NES	FDR

Hallmark IFNα Response	91	1.3	ns	−1.32	ns	1.4	ns	−0.8	ns	2.12	0.001	1.8	0.04
Bolen et al_HCV+ PBMC_UP	53	0.91	ns	−1.53	ns	1.12	ns	−1.47	0.05	1.75	0.009	1.65	0.05

**Immunosuppression gene sets**													
Hallmark Inflammatory Response	162	−1.5	ns	1.67	ns	−1.1	ns	−0.9	ns	−1.82	0.001	−2.0	0.007
Hallmark TNFα Signaling via NFκβ	184	−0.9	ns	2.05	0.002	0.8	ns	1.2	ns	−1.86	0	−2.1	0.003
Bolen et al_HCV+ PBMC_DN	56	−1.48	ns	1.78	0.02	0.8	ns	−2.38	0.005	−1.68	0.009	−1.9	0.003

**Anergic gene sets**													
Isnardi et al_ANERGIC_UP	51	1.98	0.009	1.58	0.05	1.71	0.02	1.15	ns	0.7	ns	0.97	ns
Charles et al_HCV-MC_UP	32	2.14	0.002	1.82	0.02	2.02	0	1.31	ns	0.79	ns	1.06	ns
Isnardi et al_ANERGIC_DN	26	−1.66	0.03	−1.78	0.04	−1.63	0.02	1.13	ns	−0.67	ns	−0.54	ns
Charles et al_HCV-MC_DN	32	−2.01	0.01	−2.01	0.009	−1.61	0.03	−1.21	ns	0.58	ns	−0.76	ns

FDR, false discovery rate; NES, normalized enrichment score; ns, not significant.

Ingenuity Pathway Analysis (IPA) was conducted to identify potential drivers of observed expression patterns in chronically infected samples (Figure 1E). We found that predicted drivers of upregulated genes in patients with HCV (i.e. those with a positive *Z* score) included immunosuppressive drugs, kinase inhibitors, and cytokines involved in the IFN-I response (IFNL1 and IFNA2). Conversely, predicted regulators of downregulated genes (i.e. those with a negative *Z* score) included pro-inflammatory cytokines and signaling pathways involved in driving the inflammatory response (PI3K, MAPK, and NFκβ). Once again, trends of interferon activation and immune suppression were prominent in HCV-only samples relative to other groups, but not observed in BNHL/HCV samples.

Work by Bolen and colleagues^19^ identified a set of up- and downregulated genes in PBMC from HCV-infected patients that were also enriched for interferon signaling and immune repression, respectively. We found these gene sets to be enriched in our HCV-only samples but not in BNHL/HCV samples (Figure 1F and Table 2, Figure S2D). These results demonstrate that the sustained IFN-I signaling and immunosuppression observed systemically in chronic HCV infection are also present in peripheral B cells at the transcriptional level. Importantly, these transcriptional signatures are absent or greatly reduced in samples from patients with HCV-associated BNHL, suggesting a fundamental shift in their transcriptional profile away from traditional hallmarks of chronic viral infection.

### Peripheral B cells from hepatitis C virus-associated B cell non-Hodgkin lymphoma patients display an anergic-like gene signature with evidence of clonal expansion

**Figure 2:**
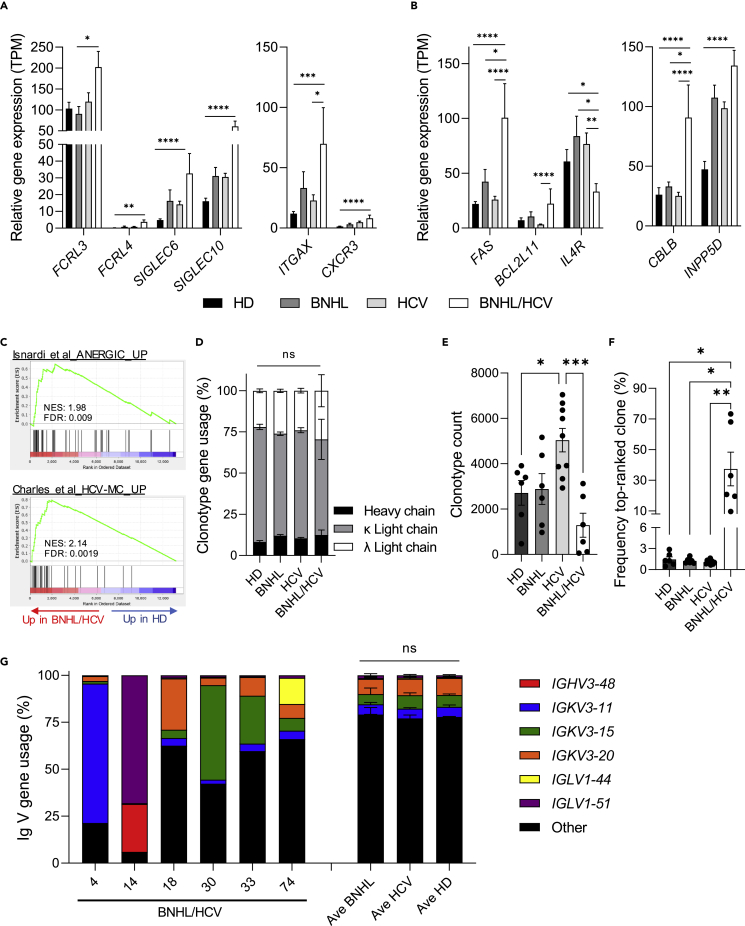
BNHL/HCV peripheral B cells display an anergic-like gene signature with evidence of clonal expansion (A and B) Relative gene expression levels, displayed as transcripts per million (TPM), for diagnosis groups. Graphs highlight select DEGs involved in inhibitory signaling (A, left), homing activity (A, right), apoptosis (B, left), and BCR signaling (B, right). Indicated p values are adjusted p values from the differential expression analysis. (C) GSEA plots of gene sets found to be upregulated in functionally anergic peripheral B cells collected from patients with autoimmune conditions (Top, Isnardi et al.^25^) and chronic HCV patients with mixed cryoglobulinemia (Bottom, Charles et al.^20^). Both plots show gene set enrichment in expressed genes (ranked by fold change) in BNHL/HCV samples vs HD. (D) Frequency of heavy or light chain gene usage among predicted clonotypes. (E) Number of clonotypes identified based on unique CDR3aa sequence. (F) Frequency of the top-ranked clone, as defined by CDR3aa sequence. (G) Frequency of total predicted variable (V) gene usage among all CDR3aa sequences for all diagnosis groups. Average gene usage is shown for BNHL, HCV, and HD groups, while BNHL/HCV samples are shown individually to highlight gene usage enrichment. All bar graphs display mean ± SEM, with individual sample values indicated by circles. FDR, false discovery rate; NES, normalized enrichment score; ns, not significant; TPM, transcripts per million. p < 0.05 ∗; p < 0.01, ∗∗; p < 0.001, ∗∗∗; p < 0.0001, ∗∗∗∗. See also Figure S3 and Table S1.

Anergic-like phenotypes consistent with those seen in autoimmune disorders have been observed in peripheral B cells from patients with HCV-associated MC (HCV-MC).^20^^,^^21^^,^^22^^,^^23^ We sought to investigate whether peripheral B cells from our patients with HCV-associated BNHL demonstrated similar anergic characteristics. Immunophenotypic properties of anergic-like B cells include increased inhibitory receptor levels on the cell surface and altered surface homing receptor expression.^21^^,^^22^^,^^23^^,^^24^^,^^25^^,^^26^ Although our data are restricted to transcriptional profiling, which may lack correlation with protein levels, we observed the upregulation of multiple anergic-associated inhibitory and homing receptors in BNHL/HCV samples (Figure 2A). This included *FCRL3/4*, *SIGLEC6/10*, *ITGAX* (CD11c), and *CXCR3*. In functional studies, anergic B cells are more prone to apoptosis and demonstrate defective BCR signaling.^20^^,^^21^^,^^22^^,^^25^^,^^26^^,^^27^ In our transcriptional analysis, we observed the upregulation of the pro-apoptotic genes *FAS* and *BCL2L11/BIM* and downregulation of the pro-survival gene *IL4R.*^28^^,^^29^ Additionally, we saw the upregulation of *CBLB* and *INPP5D* (SHIP1), which act to inhibit BCR signaling^29^^,^^30^ (Figure 2B). On a global level, we observed significant enrichment of gene sets identified in functionally anergic peripheral B cells from patients with autoimmune conditions^25^ and HCV-MC^20^ in our BNHL/HCV cohort (Figure 2C and Table 2, Figure S3A). Importantly, gene set enrichment was not observed in BNHL-only or HCV-only diagnosis groups (Table 2). These data suggest that peripheral B cells from patients with BNHL/HCV display a gene expression profile comparable to the transcriptional and functional phenotypes observed in anergic-like B cells and that this phenotype is unique to our patients with HCV-associated BNHL.

Clonal expansion is another hallmark of anergic B cell populations, with specific immunoglobulin (Ig) gene usage observed across multiple autoimmune and lymphoproliferative disorders.^20^^,^^21^^,^^26^^,^^31^ We utilized the MiXCR analysis tool^32^^,^^33^ to profile the Ig repertoire of our samples using RNA-seq data as input. MiXCR was able to align an average of 0.47% of all sequenced reads to the Ig locus, resulting in an average of 43k reads used for clonotype analysis via alignment to the complementary determining region 3 (CDR3) locus. No statistically significant difference was observed for these metrics between diagnosis groups (Figures S3B and S3C). In our analysis, predicted clonotypes, defined as unique CDR3 amino acid (CDR3aa) sequences, predominantly corresponded to the Ig light chain, specifically the kappa locus (averaging 64% of identified clonotypes across all samples), while heavy chain clonotypes comprised an average of 11% of identified clonotypes. No difference in the percentage of predicted clonotypes originating from heavy or light chain genes was observed among diagnosis groups (Figure 2D). We observed significantly more clonotypes in HCV samples compared to HD and BNHL/HCV samples (Figure 2E), which may be indicative of their chronic viral infection.

Importantly, BNHL/HCV samples displayed a high degree of clonal expansion compared to all other groups (Figure 2F). The average frequency of the top-ranked clonotypes among BNHL, HCV, and HD groups was 1-1.5%, with no individual sample exhibiting a top-ranked clone over 3%. Meanwhile, the top-ranked clone for BNHL/HCV samples had an average frequency of 37.4%, with top-ranked clones among individual samples comprising 9.6%-73.3% of observed clonotypes. Top-ranked clonotype frequency was not correlated with HCV RNA level (r = 0.6358, p value = 0.1748; Figure S3D). An investigation of top-ranked clonotypes in BNHL/HCV samples, as well as additional clonotypes reaching a frequency >10%, identified 7 unique clonotypes corresponding to 6 variable (V) gene loci (Table 3). B cell clonal expansion utilizing these V genes has been observed in a number of lymphoproliferative disorders, including HCV-associated conditions, multiple B cell lymphoma histologies, as well as autoimmune diseases, and is often associated with autoreactivity, including RF activity.^24^^,^^34^^,^^35^^,^^36^^,^^37^^,^^38^^,^^39^^,^^40^^,^^41^^,^^42^^,^^43^^,^^44^^,^^45^^,^^46^^,^^47^^,^^48^^,^^49^^,^^50^^,^^51^^,^^52^^,^^53^^,^^54^^,^^55^^,^^56^^,^^57^ Among BNHL, HCV, and HD diagnosis groups, we observed striking consistency of predicted Ig V gene usage for the 6 genes enriched in our BNHL/HCV samples (Figure 2G), demonstrating that expanded gene usage is a unique feature of BNHL/HCV peripheral B cells. Overall, we observed anergic-like properties in peripheral B cells from patients with BNHL/HCV, including gene expression changes and evidence of clonal expansion of Ig genes associated with autoimmunity, lymphoproliferation, and autoreactivity. These data support a model for HCV-associated BNHL progression that may result, in part, from clonal expansion of anergic-like B cell populations.

**Table 3:** Expanded B cell clonotypes observed in BNHL/HCV sample**s**

Sample ID	Clonotype frequency (%)^a^	CDR3aa sequence	V gene	J gene	Association with	
					Lymphoproliferative disorders	Autoimmune disorders/RF activity
A	**73.29**	CQQRRNWPPTF	*IGKV3-11*	*IGKJ2*	CLL,^43^ PVRL^34^	None
B	**68.1**	CGTWDSSLSAWVF	*IGLV1-51*	*IGLJ3*	None	None
	25.5	CAKEGVRGIDVW	*IGHV3-48*	*IGHJ3*	CLL,^50^^,^^51^ FL^49^	Celiac disease^52^
C	**33**	CQHYNNWPPWTF^b^	*IGKV3-15*	*IGKJ1*	HCV-BNHL,^39^^,^^40^^,^^41^ HCV-MC,^39^^,^^40^ HCV-CV,^24^ MALT lymphoma,^35^ pSS-MALT^36^	pSS,^45^^,^^46^ RF activity^24^
	15.1	CQQYNNWPPWTF				
D	**20.7**	CQHYNNWPPWTF^b^				
E	**19.6**	CQQHGSSPLTF	*IGKV3-20*	*IGKJ4*	CLL,^38^^,^^42^^,^^43^^,^^44^ HCV-BNHL,^39^^,^^40^^,^^41^ HCV-MC,^39^^,^^40^ HCV-CV,^24^ MALT lymphoma,^35^^,^^37^ pSS-MALT,^36^ PVRL^34^	pSS,^45^^,^^46^ RF activity,^24^^,^^47^ SLE^48^
F	**9.63**	CAAWDDSLDGVVF	*IGLV1-44*	*IGLJ2*	POEMS syndrome,^53^^,^^54^^,^^55^ systemic amyloid light amyloidosis^56^^,^^57^	None

CDR3aa, complementary determining region 3 amino acid; CLL, chronic lymphocytic leukemia; CV, cryoglobulinemia vasculitis; FL, follicular lymphoma; MALT, mucosa-associated lymphoid tissue; MC, mixed cryoglobulinemia; POEMS, polyneuropathy, organomegaly, endocrinopathy, monoclonal gammopathy, and skin changes; pSS, primary Sjӧgren’s syndrome; PVRL, primary vitreoretinal lymphoma; RF, rheumatoid factor; SLE, systemic lupus erythematosus.

a Top-ranked clonotype in each sample in bold, additional clonotypes with frequency >10% also listed.

b Shared clonotype.

### Peripheral B cells from hepatitis C virus-associated B cell non-Hodgkin lymphoma patients demonstrate an enhanced proliferative signature

**Figure 3:**
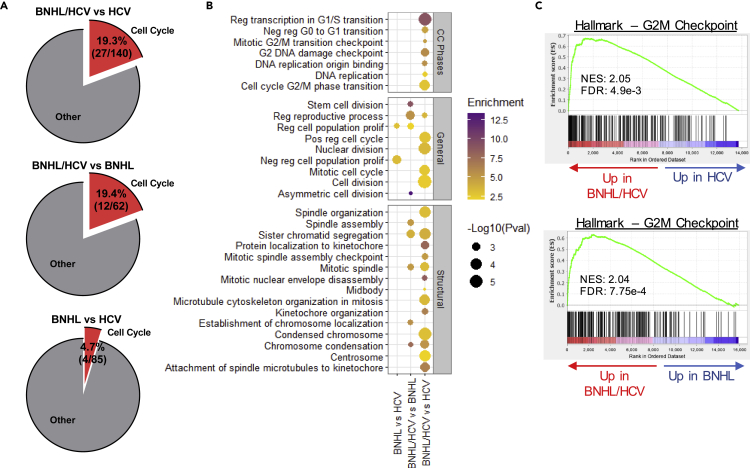
BNHL/HCV peripheral B cells demonstrate an enhanced proliferative signature (A) Pie charts illustrate the percentage of significant GO terms from analysis of upregulated DEGs that are related to the cell cycle. (B) Significant GO terms from upregulated DEGs related to cell cycle phases, general cell proliferation, and structural cell cycle terms are shown. GO terms are listed on the y axis. Circle size corresponds to GO term p value and color indicates GO term enrichment. (C) GSEA plots for expressed genes (ranked by fold change) in BNHL/HCV vs HCV (top) and BNHL (bottom) comparisons. FDR, false discovery rate; NES, normalized enrichment score. See also Figure S4 and Table S2.

To further investigate transcriptional signatures of BNHL/HCV samples, we examined DEGs among dual diagnosis patients relative to HCV- and BNHL-only samples. We observed an enrichment of cell cycle-related GO terms among DEGs upregulated in BNHL/HCV samples relative to either condition alone, and this trend was not observed in BNHL samples compared to HCV-only samples (Figure 3A, Table S2). Specifically, we observed the enrichment of GO terms related to cell cycle phases, general proliferation, and structural changes essential for proper cell division (Figure 3B). We observed strong enrichment of the Hallmark—G2M Checkpoint gene set in BNHL/HCV comparison groups (Figure 3C), with no significant enrichment observed in BNHL vs HCV (NES = 0.92, FDR = 0.71). Interestingly, GSEA also identified significant enrichment of multiple gene sets associated with proliferative cancer subtypes (Figure S4). These spanned cancer histologies, including both blood and epithelial cancers, and etiologies, including non-viral- and viral-associated cancers. The latter included HPV+ cervical cancer and HCV+ hepatocellular carcinoma. Our bioinformatic analyses, therefore, indicate that peripheral B cells from patients with BNHL/HCV were enriched in the expression of proliferation-associated genes and that this proliferative transcriptional phenotype may be a unique feature of the dual diagnosis condition relative to non-viral-associated BNHL.

### Upregulation of epigenetic regulatory genes is a common feature among lymphoma samples and strongly correlates with hepatitis C virus-associated B cell non-Hodgkin lymphoma clonal expansion

**Figure 4:**
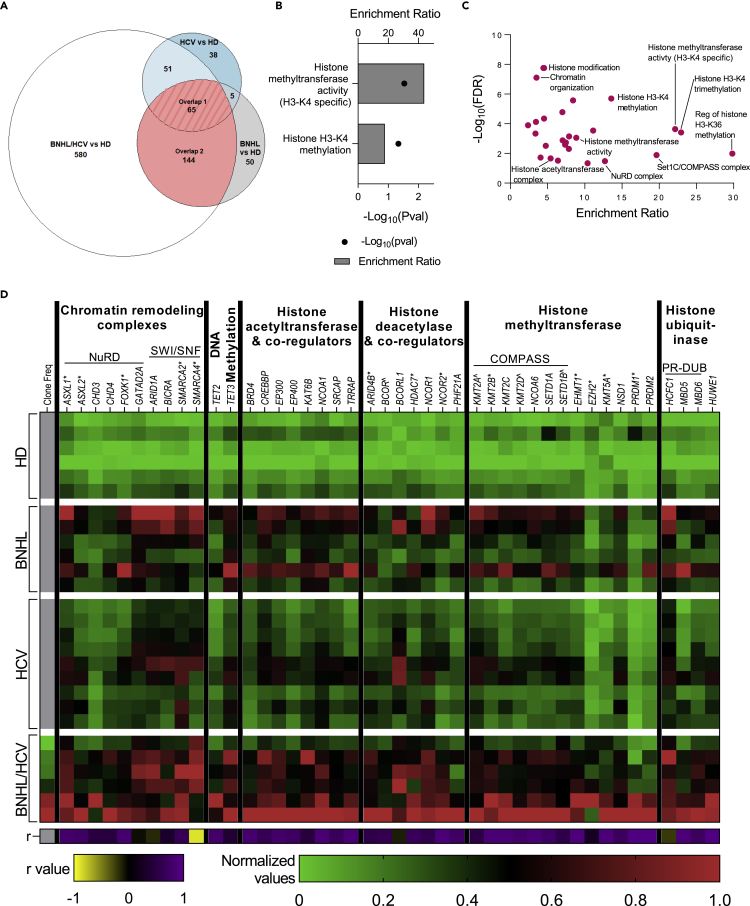
Upregulation of epigenetic regulatory genes is a common feature among lymphoma samples and strongly correlates with BNHL/HCV clonal expansion (A) Area-proportional Venn diagram of overlapping DEGs among BNHL, BNHL/HCV, and HCV samples versus HD. Overlap 1 (pink stripes) includes the 65 genes common to all 3 comparisons, and Overlap 2 (pink) includes the 209 genes common to BNHL and BNHL/HCV comparisons. (B) Significantly enriched epigenetic GO terms from analysis of Overlap 1 genes. (C) Significantly enriched epigenetic GO terms from analysis of Overlap 2 genes. Select terms are labeled. (D) Heatmap displays column-normalized gene expression values (green to red legend) for select genes annotated to epigenetic GO terms identified in C. Genes are grouped based on their epigenetic activity and involvement in multi-subunit regulatory complexes. Genes marked with ˆ correspond to Overlap 1, unmarked genes correspond to Overlap 2, and those marked with ∗ are upregulated only in BNHL/HCV. For BNHL/HCV samples, the left most column indicates the normalized frequency of the top-ranked clonotype. The Pearson correlation coefficient (r) between clonotype frequency and gene expression is shown in the bottom row (yellow to purple legend). See also Table S2.

To identify common biological pathways and processes altered in peripheral B cells across disease conditions, we investigated overlapping DEGs among BNHL, BNHL/HCV, and HCV samples relative to HD. We observed 65 genes to be upregulated in all 3 comparison groups (Overlap 1) and an additional 144 genes upregulated in both the BNHL/HCV and BNHL comparison groups (Overlap 2) (Figure 4A). GO analysis of Overlap 1 genes revealed strong enrichment of histone methylation-related GO terms (Figure 4B, Table S2). In particular, we observed the upregulation of genes comprising the COMPASS complex (*KMT2A*, *KMT2D*, *SETD1B*), a transcriptional activator that adds mono-, di-, or trimethylation marks to H3K4,^58^ as well as the transcriptional repressor, BCOR. GO analysis of Overlap 2 genes (n = 209) similarly found significant enrichment of epigenetic-related GO terms (Figure 4C, Table S2). An investigation of the DEGs annotated to these GO terms identified genes involved in multiple epigenetic regulatory complexes, including the aforementioned COMPASS complex, the NuRD and SWI/SNF chromatin remodeling complexes, and the PR-DUB deubiquitinase complex. Furthermore, we observed the upregulation of a number of well-known histone acetyltransferases and deacetylases and their associated co-activators/repressors (*BRD4, CREBBP, EP300, EP400, KAT6B, NCOR1*), as well as the DNA demethylases *TET2/3.* Additional epigenetic regulators and complex family members were upregulated exclusively in BNHL/HCV samples (*ASXL1/2, HDAC7, NCOR2, KMT2B/5A, EHMT1, EZH2*). To investigate gene expression changes in the context of clonotype expansion, BNHL/HCV gene expression was examined with respect to its correlation to top-ranked clonotype frequency, and we observed a remarkably strong positive correlation (Figure 4D). While we observed the upregulation of epigenetic modifying genes across all 3 diagnosis groups, BNHL and especially BNHL/HCV samples demonstrated an even more profound enrichment of epigenetic upregulation. This indicates epigenetic regulatory processes are more active in peripheral B cells from patients with lymphoma, and suggests the activation of these processes may be a shared mechanism driving viral- and non-viral-associated lymphoma progression. The strong correlation of epigenetic gene expression with the observed degree of clonotype expansion in BNHL/HCV samples suggests epigenetic mechanisms are also associated with peripheral B cell anergy. This presents the intriguing hypothesis that epigenetic regulatory mechanisms may be involved in regulating the anergic state.

## Discussion

Clinical and epidemiological evidence demonstrate an association between HCV infection and BNHL^4^^,^^5^^,^^59^; however, the biological mechanisms underlying this association are incompletely understood. In an effort to further elucidate this relationship, we performed RNA-seq on peripheral B cells from patients with BNHL alone, HCV infection alone, and patients with HCV-associated BNHL. By investigating DEGs among these diagnosis groups relative to HD and each other, we sought to identify unique and shared mechanisms by which HCV infection promotes BNHL development.

Analysis of DEGs in patients with HCV infection alone identified a strong IFN-I signature, driven by the upregulation of ISGs. We also observed gene expression changes consistent with a general repression of immune activity. IFN-I signaling is induced in response to viral infection and, in the context of chronic infection, its sustained activity has been shown to promote infection and suppress the immune response.^60^ Our observations are, therefore, consistent with the literature; however, transcriptional signatures of chronic HCV infection have generally been observed in analyses of heterogeneous PBMC populations.^19^ Identification of this signature in circulating B cells suggests B cells, themselves, are specifically affected by chronic infection, although the mechanisms behind these transcriptional alterations remain unknown and may result from direct and/or indirect contact with HCV. Importantly, gene signatures of IFN-I and immunosuppression were absent or greatly reduced in patients with HCV-associated BNHL. Therefore, peripheral B cells in patients with BNHL/HCV displayed a transcriptional shift away from traditional expression patterns of chronic infection.

The development of HCV-associated BNHL is often, but not always, preceded by other HCV-LPDs, such as MC or systemic vasculitis, and B cells from patients with HCV-LPDs have demonstrated anergic-like phenotypes.^20^^,^^21^^,^^59^ B cell anergy is a reversible dysfunctional state induced during B cell development to mitigate the negative effects of autoreactive B cells. Anergic B cells remain in the periphery but are unresponsive to BCR stimulation; however, failure to maintain the anergic state can result in autoimmunity and lymphoproliferation.^23^^,^^25^^,^^27^^,^^31^ We observed an anergic-like transcriptional profile in peripheral B cells from patients with BNHL/HCV. This included overexpression of inhibitory and homing receptors, pro-apoptotic genes, and BCR signaling inhibitors. Furthermore, we saw the enrichment of anergic-like gene sets identified in anergy-enriched B cell populations from patients with autoimmune diseases and HCV-MC.^20^^,^^25^ Future studies will reveal whether the transcriptional changes we have observed in peripheral B cells reflect functional anergic phenotypes, including elevated apoptosis and impaired responses to receptor-mediated stimulation.

The anergic state is thought to have evolved so as to maintain BCR diversity, in particular, due to the propensity for foreign pathogens to employ molecular mimicry to evade immune detection.^31^^,^^61^ This is supported by the observation of B cell expansion, following viral infection, of clones expressing BCRs derived from Ig genes possessing both anti-viral and self-antigen reactivity.^31^^,^^40^^,^^61^^,^^62^^,^^63^ Expanded usage of these same Ig genes is often found in anergic B cell populations.^20^^,^^21^^,^^22^^,^^31^ We investigated Ig gene expression in our cohort using BCR repertoire analysis of RNA-seq data. We observed that the extent of mappable Ig gene transcripts and predicted heavy and light chain distribution was consistent among diagnosis groups, suggesting any biases in our analysis method were uniform. Thus, although not an exhaustive investigation of the BCR repertoire, with this method we were able to adequately capture clonotype overrepresentation.^33^^,^^64^ We observed significant clonal expansion in peripheral B cells from patients with BNHL/HCV, and expanded clonotypes were predicted to derive from 6 Ig variable genes. This included *IGKV3-15* and *IGKV3-20*, whose overrepresentation has been well-studied in multiple conditions, including non-viral- and viral-associated lymphomas and autoimmune diseases, and whose encoded BCRs have been shown to possess autoreactivity.^24^^,^^34^^,^^35^^,^^36^^,^^37^^,^^38^^,^^39^^,^^40^^,^^41^^,^^42^^,^^43^^,^^44^^,^^45^^,^^46^^,^^47^^,^^48^ Indeed, the specific *IGKV3-15* CDR3aa sequences we observed in our patients with BNHL/HCV were observed in patients with Sjӧgren’s syndrome-associated MALT lymphoma in a study by Bende et al.*,*^36^ where they observed the Q106H mutation, found in our patients (see Table 3), to enhance RF activity. The other expanded clonotypes we observed in our patient population (*IGKV3-11, IGHV3-48*, and *IGLV1-44*) have been associated with lymphoproliferative disorders, autoimmunity, and multiple B cell lymphoma histologies,^34^^,^^43^^,^^50^^,^^51^^,^^52^^,^^53^^,^^54^^,^^55^^,^^56^^,^^57^ including CLL, which has, itself, been shown to display anergic properties.^65^^,^^66^ Our observation of *IGLV1-55-*derived clonotype expansion appears to be the first instance of its association with BNHL in the literature. Studies of lymphoma-associated clonal expansion are typically conducted on B cells isolated from lymphoma tissue. Our detection of clonal expansion in peripheral B cells of patients with BNHL/HCV represents an exciting observation, and future studies will determine if peripheral clonotype composition reflects that found at the tumor site. If so, peripheral clonal expansion may represent a useful biomarker of disease development.

Our data support an indirect mechanism of HCV-mediated B cell transformation driven by clonal expansion of anergic-like B cells. In the context of chronic viral infection, it is thought that excessive viral stimulation can overcome anergic hurdles and allow for B cell activation and germinal center (GC) activity^31^; however, it is unclear what mechanisms support anergic escape. In our BNHL/HCV samples, we observed significant upregulation of epigenetic regulatory genes that were positively correlated with the degree of clonal expansion, suggesting epigenetic mechanisms may be involved in regulating the anergic state. We also observed the enrichment of a proliferative transcriptional signature in peripheral BNHL/HCV B cells. While this observation is consistent with the clonal expansion observed in the periphery, it is at odds with the impaired proliferation typical of anergic cells.^27^ In this way, peripheral B cells may represent an intermediary or transitional phenotype between HCV-specific anergic B cells in the periphery and lymphoma cells at the tissue site. An investigation of matched tissue samples will determine whether peripheral transcriptional signatures and clonal expansion are reproduced in lymphoma tissue and may shed light on the relationship of the peripheral phenotypes to lymphoprogression. These data present the intriguing hypothesis that the epigenetic regulation of proliferation is involved in maintaining the anergic state and that the dysregulation of these processes could be involved in anergic escape and lymphoma progression.

B cells from patients with non–viral-associated BNHL also demonstrated an upregulation of epigenetic regulatory genes. As these samples did not display an anergic transcriptional signature, if epigenetic mechanisms are regulating anergy in BNHL/HCV peripheral B cells, they are likely performing another function in BNHL-only samples. Within GCs, epigenetic proteins are crucial for regulating the transcriptional changes needed for dark and light zone function, and mutations in epigenetic regulatory genes are a hallmark of many B cell lymphoma subtypes.^67^^,^^68^ We observed many epigenetic genes commonly mutated in B cell lymphomas to be upregulated in our cohort. This includes *CREBBP, EP300, EZH2*, and *KMT2D*, which are mutated in 95% of FL and 50% of DLBCL cases.^68^ These proteins have epigenetic regulatory functions in GC B cells, including the regulation of proliferation and BCL6 activity. Furthermore, BCL6 interactions with co-repressor proteins BCOR and NCOR1/2, which were differentially expressed in our dataset, represent another key regulator of GC B cell function that is dysregulated in B cell lymphomas.^67^^,^^68^^,^^69^ In this way, many of the epigenetic regulatory genes upregulated in our BNHL and BNHL/HCV samples have significant roles in lymphoma development.

The data presented in this study support epigenetic dysfunction as a possible shared mechanism driving lymphoprogression in viral- and non-viral-associated lymphoma. Furthermore, the relationship between epigenetic regulation and B cell anergy, observed in peripheral B cells from patients with HCV-associated BNHL, has clinical relevance not only in lymphomagenesis, but also in the development and treatment of autoimmunity.

### Limitations of the study

This study was conducted on a limited number of patient samples, and we observed variation in BNHL subtypes between our BNHL-only and HCV-associated lymphoma groups. Despite differences in subtype classification, both lymphoma cohorts included a mix of low-grade (Stage I/II) and high-grade (Stage III/IV) lymphomas, although the limited sample number precluded analyses segregated by subtype or stage. Our results support future studies with an expanded cohort to investigate the influence these factors may have on peripheral B cell transcriptomics. Additionally, gene expression differences we have observed may not translate to differences in protein levels or cell function. Functional studies of peripheral B cells will be required to address this concern.

## STAR★Methods

### Key resources table

**Table:** 

REAGENT or RESOURCE	SOURCE	IDENTIFIER
**Antibodies**		

Human CD19 clone HIB19	BD Biosciences	Cat# 555413; RRID: AB_395813
Human CD14 clone M5E2	BD Biosciences	Cat# 555397; RRID: AB_395798
Human CD15 clone W6D3	BD Biosciences	Cat# 563141; RRID: AB_2738025
Human CD3 clone SK7	BD Biosciences	Cat# 563797; RRID: AB_2744383
Human CD56 clone B159	BD Biosciences	Cat# 557747; RRID: AB_396853
Human CD45 clone 2D1	BD Biosciences	Cat# 557833; RRID: AB_396891

**Biological samples**		

Human peripheral blood mononuclear cells (PBMCs)	Liver Center, Ulaanbaatar, Mongolia	N/A

**Critical commercial assays**		

Xpert HCV Viral Load	Cepheid	Cat# GXHCV-VL-CE-10
OnSite HBsAg Rapid Test	CTK Biotech	Cat# R0040C
OnSite HCV Ab Plus Rapid Test	CTK Biotech	Cat# R0023S
EasySep Human B Cell Isolation Kit	StemCell Tech	Cat# 17954
RNeasy Plus Micro kit	Qiagen	Cat# 74034
7-AAD Viability Stain Kit	BioLegend	Cat# 420403
TruSeq RNA Library Prep for Enrichment	Illumina	Cat# 20020189
TruSeq RNA Enrichment	Illumina	Cat# 20020490
Illumina Exome Panel	Illumina	Cat# 20020183
KAPA Library Quantification Kit	Roche	Cat# 07960336001

**Deposited data**		

RNA sequencing data files	This paper	GEO: GSE215876

**Software and algorithms**		

FlowJo v10	FlowJo, LLC	https://www.flowjo.com/
BaseSpace RNA-Seq Alignment App	Illumina	https://www.illumina.com/products/by-type/informatics-products/basespace-sequence-hub/apps/rna-seq-alignment.html
R package DESeq2	Bioconductor	https://bioconductor.org/packages/release/bioc/html/DESeq2.html
BaseSpace MiXCR Immune Repertoire Analyzer App (no longer available on BaseSpace)	Bolotin et al.^31^	https://github.com/milaboratory/mixcr/releases/tag/v4.0.0
R package topGO	Bioconductor	https://bioconductor.org/packages/release/bioc/html/topGO.html
Web-based Gene Set Analysis Toolkit (WebGestalt)	Wang et al.^68^	http://www.webgestalt.org/
Gene Set Enrichment Analysis (GSEA)	Subramanian et al.^69^	https://www.gsea-msigdb.org/gsea/index.jsp
Ingenuity Pathway Analysis (IPA)	Qiagen	https://digitalinsights.qiagen.com/products-overview/discovery-insights-portfolio/analysis-and-visualization/qiagen-ipa/
Prism 9	GraphPad Software	https://www.graphpad.com/

### Resource availability

#### Lead contact

Further information and requests for resources and reagents should be directed to and will be fulfilled by the lead contact, Valeria De Giorgi (valeria.degiorgi@nih.gov).

#### Materials availability

This study did not generate new unique reagents.

### Experimental model and subject details

#### Human subjects and ethics statement

Patients were recruited from the First Central Hospital of Mongolia (Ulaanbaatar, Mongolia) in accordance with study approval provided by the Ethics Committee of the Ministry of Health (No.4, approval date 6/19/2017). A total of 27 patients were included in this study, consisting of individuals diagnosed with B cell non-Hodgkin’s lymphoma without viral infection (n = 6), chronic HCV infection without lymphoma (n = 9), HCV-associated B cell non-Hodgkin’s lymphoma (n = 6), and healthy donors (n = 6). Information on age and sex of participants can be found in Table 1. The influence of sex and age on the results of this study were not investigated. All patients provided written informed consent, and samples were de-identified prior to distribution.

### Method details

#### Clinical testing and evaluation of patient samples

BNHL diagnosis was determined following cytological or pathological examination according to the Revised European-American Lymphoma classification and the WHO Classification of Tumors of Hematopoietic and Lymphoid Tissues. Blood collection from lymphoma patients occurred within 6 months of their diagnosis, with most collections occurring within 1 month, and all patients had not received treatment for their lymphoma at time of sample collection. Routine clinical blood tests were performed at diagnostic facilities at the First Central Hospital of Mongolia, and viral testing was performed at the Liver Center (Ulaanbaatar, Mongolia). HCV RNA quantification was performed using the Xpert HCV Viral Load test on the fully-automated, closed system GeneXpert IV and XVI platforms (Cepheid), and HBV surface antigen and anti-HCV antibody detection were performed using the OnSite HBsAg Rapid Test and HCV Ab Plus Rapid Test (CTK Biotech), respectively, according to manufacturer’s instructions.

#### PBMC sample collection and storage

Initial sample collection and processing was performed by research collaborators in Mongolia at the Liver Center. PBMC were isolated from whole blood via density centrifugation using Ficoll-Paque Plus solution (GE Healthcare). Whole blood was overlaid onto Ficoll-Paque plus and centrifuged at 1350rpm for 30 min at room temperature with the brake off. The interphase layer containing PBMC was then collected and washed at 400x g for 10 min at room temperature, followed by 2 additional washes at 200x g for 10 min at room temperature. PBMC were stored in freezing media, consisting of 40% FBS, 52% RPMI, and 8% DMSO. Samples were stored at −80°C until time of transport, as liquid nitrogen storage was unavailable. For transport to NIH facilities, samples were shipped on dry ice in a temperature-controlled and monitored container. Upon receipt of samples, PBMC were stored in liquid nitrogen until further processing.

#### PBMC processing and B cell isolation

PBMC samples were thawed in a 37°C water bath, and cells were immediately and gently added to pre-chilled thaw diluent (1:30 ratio) containing Plasma-Lyte A Injection, pH 7.4 (Baxter Healthcare Corp) with added heparin (10 IU/mL, StemCell Tech) and DNase I (0.01 mg/mL, Stem Cell Tech). Cells were centrifuged at 300x g for 5 min at room temperature and pellet resuspended in 1X RoboSep buffer (StemCell Tech) for subsequent B cell isolation. If cell aggregates were observed, cell suspensions were filtered with a 37 μm cell strainer (StemCell Tech). B cell isolation was performed using the EasySep Human B cell Isolation kit (StemCell Tech) with minor adjustments to the standard protocol, including performing isolation in a 96-well plate with proportionately scaled-down reagents. B cell purity was assessed via flow cytometry for select samples. Cells were stained with: CD19 PE (BD; clone HIB19), CD14 FITC (BD; clone M5E2), CD15 BV510 (BD; clone W6D3), CD3 BV421 (BD; clone SK7), CD56 PE-Cy7 (BD; clone B159), CD45 APC-Cy7 (BD; clone 2D1), and 7-AAD (BioLegend). Samples were acquired on a CytoFlex analyzer (Beckman Coulter), and data were analyzed using FlowJo v10 (FlowJo, LLC).

#### RNA isolation, library preparation and sequencing

RNA was isolated from B cells using the RNeasy Plus Micro kit (Qiagen) according to the manufacturer’s protocol. RNA was quantified on a NanoDrop 2000 (Thermo Scientific) and assessed for quality via RNA integrity number (RIN) on an Agilent 2100 Bioanalyzer (Agilent Technologies). Libraries were prepared using the TruSeq RNA Exome kit (Illumina), following manufacturer’s protocol. Library quality and size were assessed using an Agilent 2100 Bioanalyzer (Agilent Technologies), and KAPA Library Quantification Kits (Roche) were used for qPCR-based library quantification. Libraries were normalized to 10 nM and pooled for sequencing on NextSeq 500/550 High Output flow cells (v2.5; Illumina) on a NextSeq 550 instrument (Illumina).

#### Bioinformatic analysis of RNA-seq data

Quality metrics for sequenced libraries and library alignment were performed using the BaseSpace Sequencing Hub (Illumina), a cloud-based data analysis platform. Reads were aligned using the RNA-Seq Alignment application (v2.0.2, hg19 reference genome). This application utilized the STAR aligner, and transcript quantification was performed using Salmon. These analyses were conducted using the default parameters. Sequencing files are available on the GEO repository: GSE215876. Differential expression analysis was performed in R (v4.0.2) using the DESeq2 package (v1.28.1). The DESeqDataSet was designed to test for the effect of sample diagnosis group while controlling for flow cell batch effects (design = ∼Batch + Diagnosis). Genes were considered differentially expressed if they had a log2 fold change < -1 or >1 and a Benjamini-Hochberg adjusted p-value of <0.05. BCR CDR3 repertoire analysis was performed in BaseSpace using the MiXCR Immune Repertoire Analyzer application (v2.1.11)^32^^,^^33^ with default settings for RNA-seq input data.

Gene ontology analysis was performed with the topGO R package (v2.40.0) and the web-based WebGestalt tool (www.webgestalt.org).^70^ GSEA analysis was performed using the desktop application (v4.0.3)^71^, and additional data analysis was performed using the Ingenuity Pathway Analysis tool (Qiagen).

### Quantification and statistical analysis

Statistical analyses were conducted in GraphPad Prism (GraphPad, v9.3.1). Analyses consisting of two groups were performed using an unpaired T test. Analyses of more than 2 groups were performed using either one-way ANOVA with Tukey’s correction for multiple comparisons or the non-parametric Kruskal-Wallis test followed by Dunn’s multiple comparison testing. Strength of correlation was determined using Pearson correlation coefficient (R value). Details of statistical parameters can be found in the figure legends. A p value of <0.05 was considered statistically significant (p < 0.05 ∗; p < 0.01, ∗∗; p < 0.001, ∗∗∗; p < 0.0001, ∗∗∗∗). Figures were created using GraphPad Prism (v9.3.1) and R, and the graphical abstract was created with BioRender.com.

## Data Availability

• RNA sequencing data generated in this study have been deposited in the NIH Gene Expression Omnibus and are publicly available under the accession ID GSE215876. • This paper does not report original code. • Any additional information required to reanalyze the data reported in this paper is available from the lead contact upon request.

## Supplemental information

Document S1. Figures S1–S4

Table S1. Differentially expressed gene lists, related to Figures 1, 2, S1, and S2

Table S2. Gene ontology analyses, related to Figures 1, 3, 4, and S2
