# Supplementary material for: Peripheral B cells from patients with hepatitis C virus-associated lymphoma exhibit clonal expansion and an anergic-like transcriptional profile
Source: iScience. 2022 Dec 15;26(1):105801. doi: 10.1016/j.isci.2022.105801 (PMC9813790; doi:10.1016/j.isci.2022.105801)
Supplement: Document S1. Figures S1–S4 [file mmc1.pdf]

iScience, Volume *26*

Supplemental information

Peripheral B cells from patients with hepatitis C virus-associated lymphoma exhibit clonal expansion and an anergic-like transcriptional profile

Amanda N. Henning, Myagmarjav Budeebazar, Delgerbat Boldbaatar, Dahgwahdorj Yagaanbuyant, Davaadorj Duger, Khishigjargal Batsukh, Huizhi Zhou, Ryan Baumann, Robert D. Allison, Harvey J. Alter, Naranjargal Dashdorj, and Valeria De Giorgi

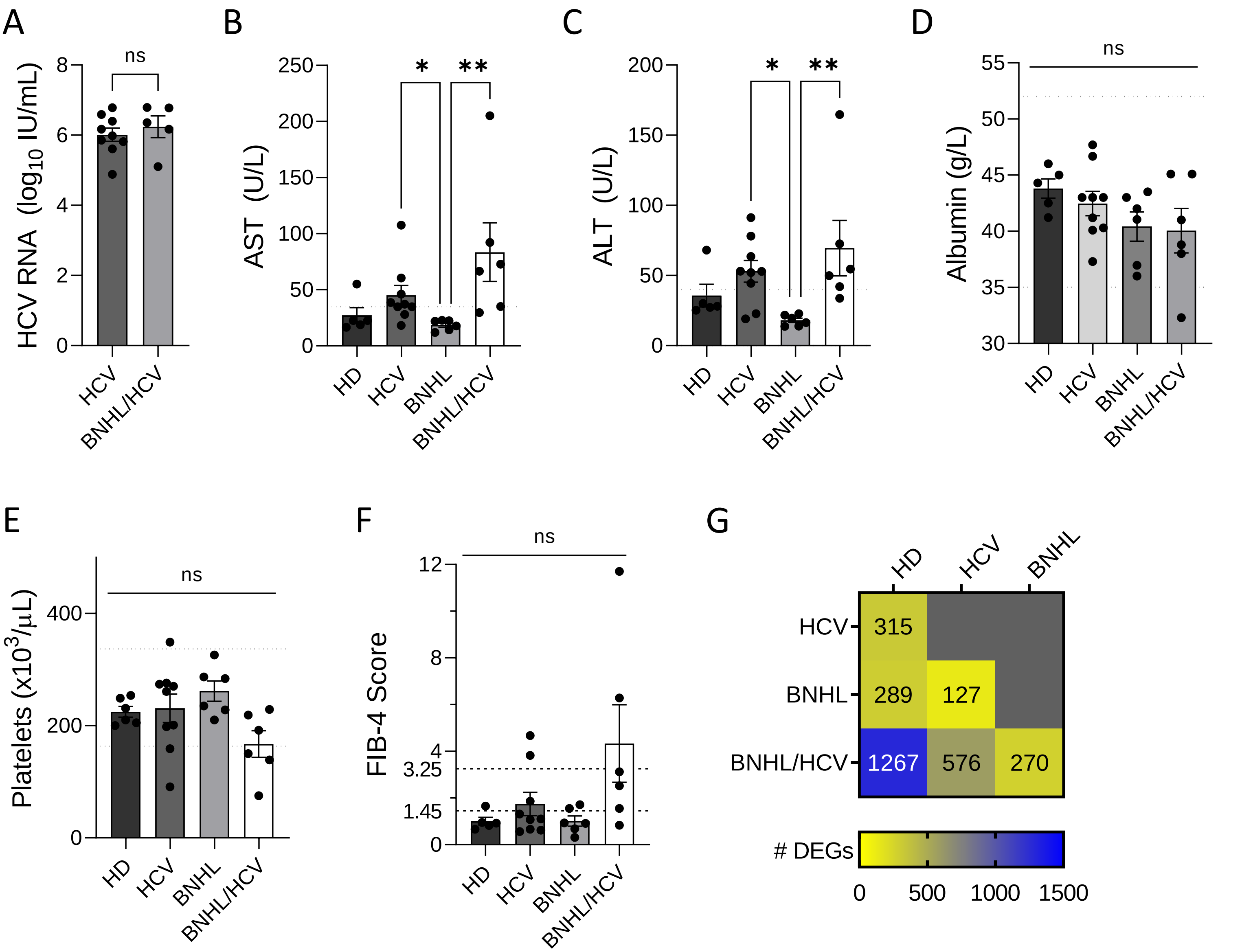

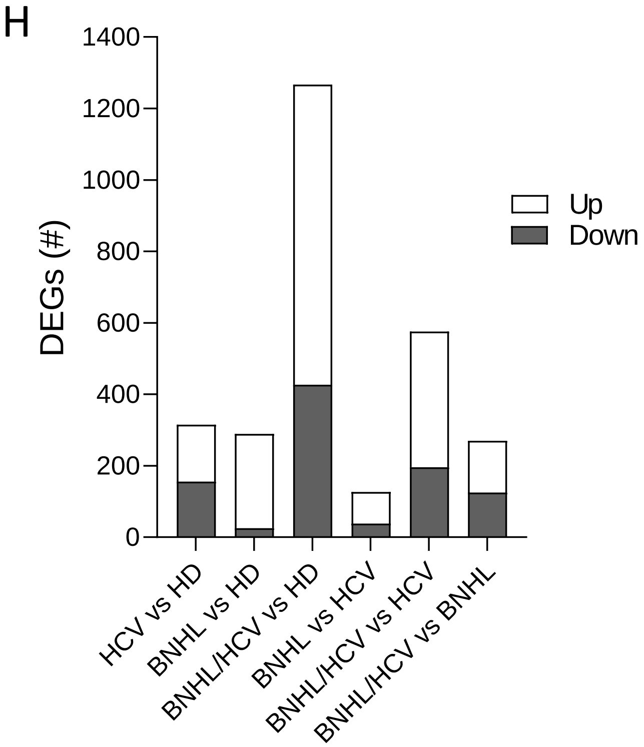

Figure S1 – Clinical characteristics of patient cohorts and transcriptional analysis of peripheral B cells. Related to Table 1 and Table S1. A) HCV viral load is quantified for HCV (n=9) and BNHL/HCV (n=5) groups. **B-E)** Patient blood samples were assessed for levels of AST (B), ALT (C), albumin (D), and platelets (E) in HD (n=6 platelets, n=5 AST/ALT/albumin), HCV (n=9), BNHL (n=6), and BNHL/HCV (n=6) groups. Normal ranges are indicated by the dotted lines. **F)** FIB-4 scores were calculated according to the formula (Age*AST)/(Platelets*√ALT). A score <1.45 suggests an absence of advances fibrosis, while a score >3.25 suggests the presence of advanced disease (dashed lines; HD, n=5; HCV, n=9; BNHL, n=6; BNHL/HCV, n=6). **G.** Heatmap indicating number of differentially expressed genes (DEGs) between each comparison group. **H.** Stacked bar graph indicates the number of upregulated (white) and downregulated (grey) DEGs in each comparison group. DEGs are defined as having a log2 fold change <-1 or >1 and an adjusted p-value <0.05. All bar graphs display mean ±SEM, with individual patient values indicated by circles. AST, aspartate aminotransferase; ALT, alanine transaminase; HD, healthy donor; ns, not significant. p<0.05 *; p<0.01, **

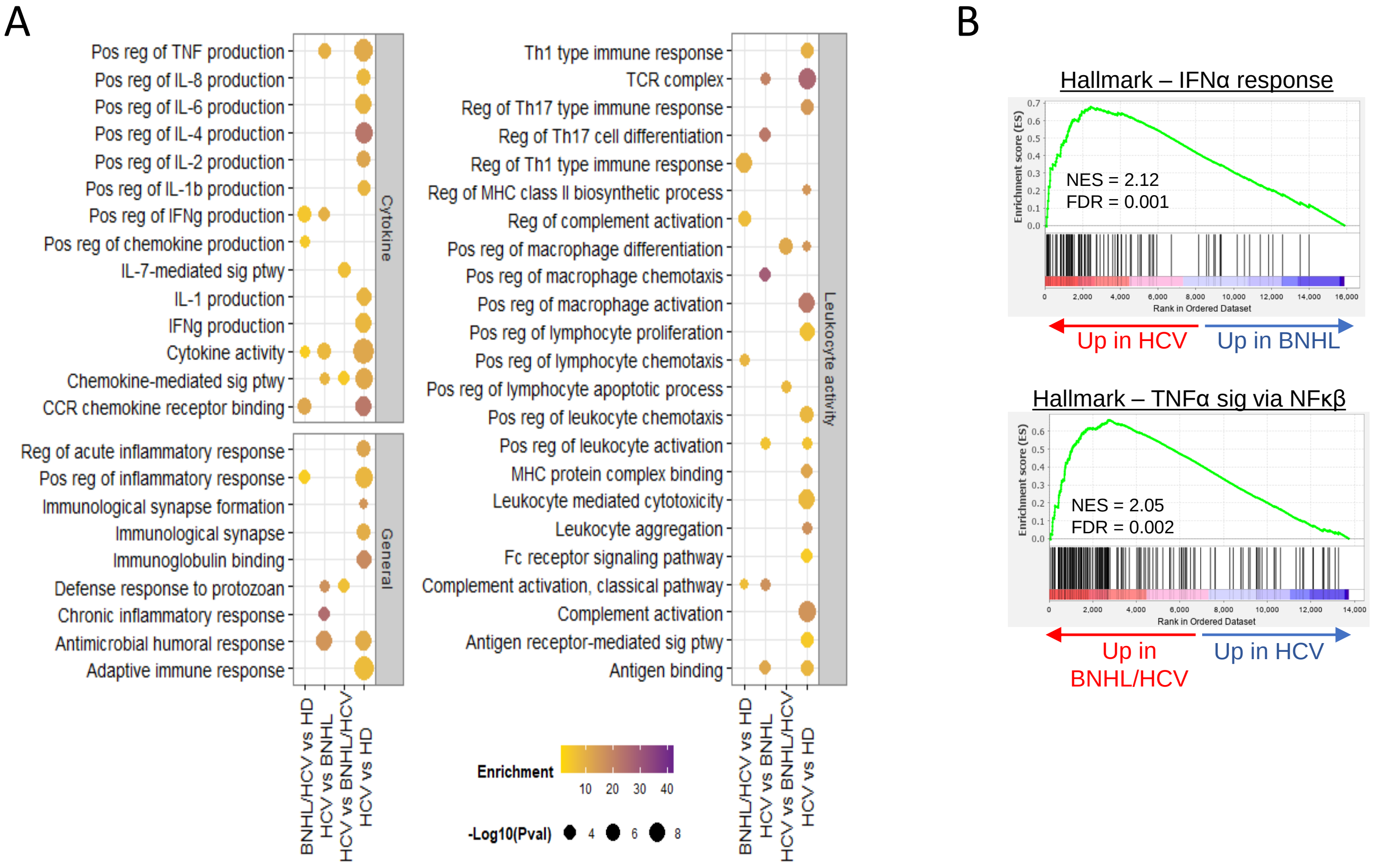

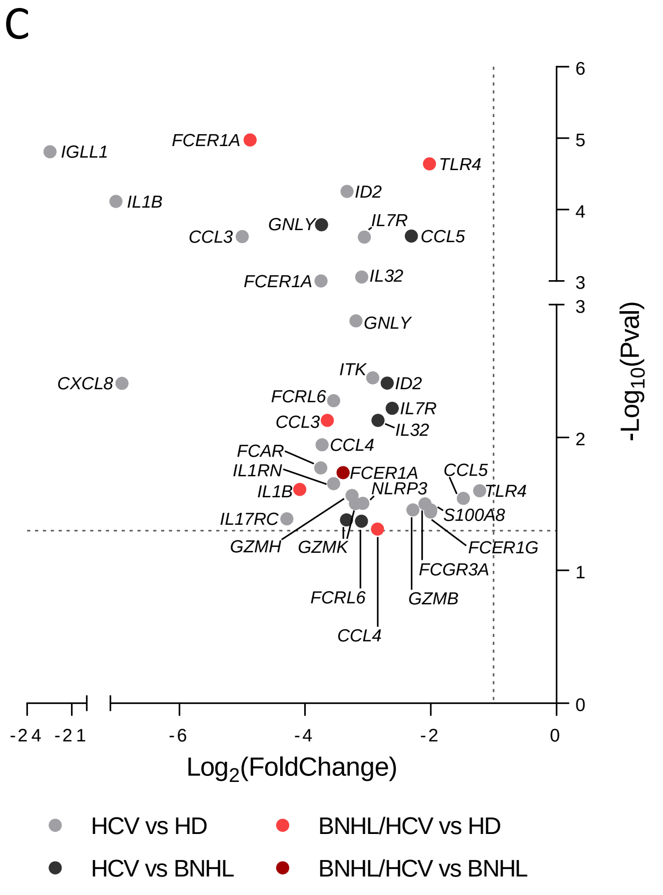

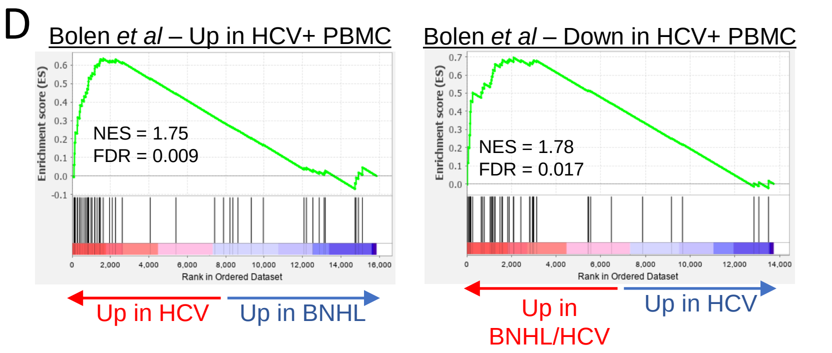

Figure S2 – Transcriptional signatures associated with chronic HCV are altered in HCV-associated BNHL. Related to Figure 1, Table 2, Table S1, and Table S2. A) All significant GO terms from downregulated DEGs in HCV infected groups related to cytokine activity, leukocyte activity, and general immune response. GO terms are listed on the y-axis. Circle size corresponds to GO term p-value and color indicates GO term enrichment. **B)** GSEA plots for expressed genes (ranked by fold change) in HCV vs BNHL (top) and BNHL/HCV vs HCV (bottom) comparisons. **C)** Select DEGs among HCV and BNHL/HCV comparison groups involved in inflammatory processes. Log2 fold change is listed on the x-axis and –log10(adjusted p-value) is on the y-axis. Dashed lines indicate DEG cutoffs. **D)** GSEA plots of gene sets found to be up- and downregulated (left and right, respectively) in PBMC The left plots collected from chronic HCV patients in a study by Bolen et al. [1]. shows gene set enrichment in expressed genes (ranked by fold change) in HCV vs BNHL samples, and the right corresponds to the BNHL/HCV vs HCV comparison group. FDR, false discovery rate; NES, normalized enrichment score

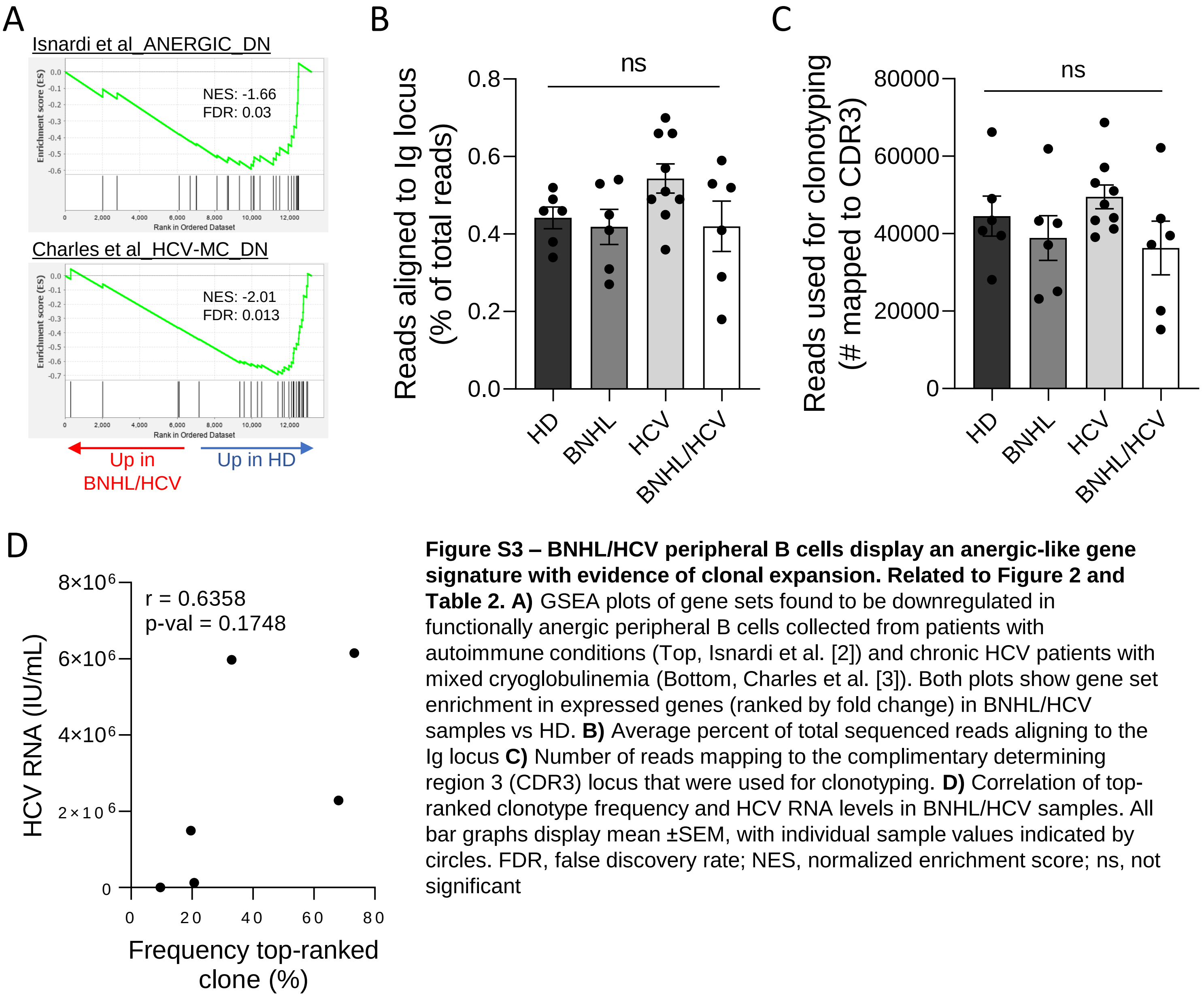

Figure S3 – BNHL/HCV peripheral B cells display an anergic-like gene signature with evidence of clonal expansion. Related to Figure 2 and Table 2. A) GSEA plots of gene sets found to be downregulated in functionally anergic peripheral B cells collected from patients with autoimmune conditions (Top, Isnardi et al. [2]) and chronic HCV patients with mixed cryoglobulinemia (Bottom, Charles et al. [3]). Both plots show gene set enrichment in expressed genes (ranked by fold change) in BNHL/HCV samples vs HD. **B)** Average percent of total sequenced reads aligning to the Ig locus **C)** Number of reads mapping to the complimentary determining region 3 (CDR3) locus that were used for clonotyping. **D)** Correlation of top-ranked clonotype frequency and HCV RNA levels in BNHL/HCV samples. All bar graphs display mean ±SEM, with individual sample values indicated by circles. FDR, false discovery rate; NES, normalized enrichment score; ns, not significant

Gene set name/description

GRAHAM_CML_DIVIDING_VS_NORMAL_QUIESCENT_UP	ROSTY_CERVICAL_CANCER_PROLIFERATION_CLUSTER	CHIANG_LIVER_CANCER_SUBCLASS_PROLIFERATION_UP	ZHAN_MULTIPLE_MYELOMA_PR_UP
Genes upregulated in dividing vs quiescent $CD34^{+}$ cells from CML patients	Genes in HPV+ cervical cancer positively correlated with E6/E7 expression	Genes upregulated in a proliferative subclass of HCV+ HCC	Genes upregulated in cluster of MM characterized by increased expr of proliferation/cell cycle genes

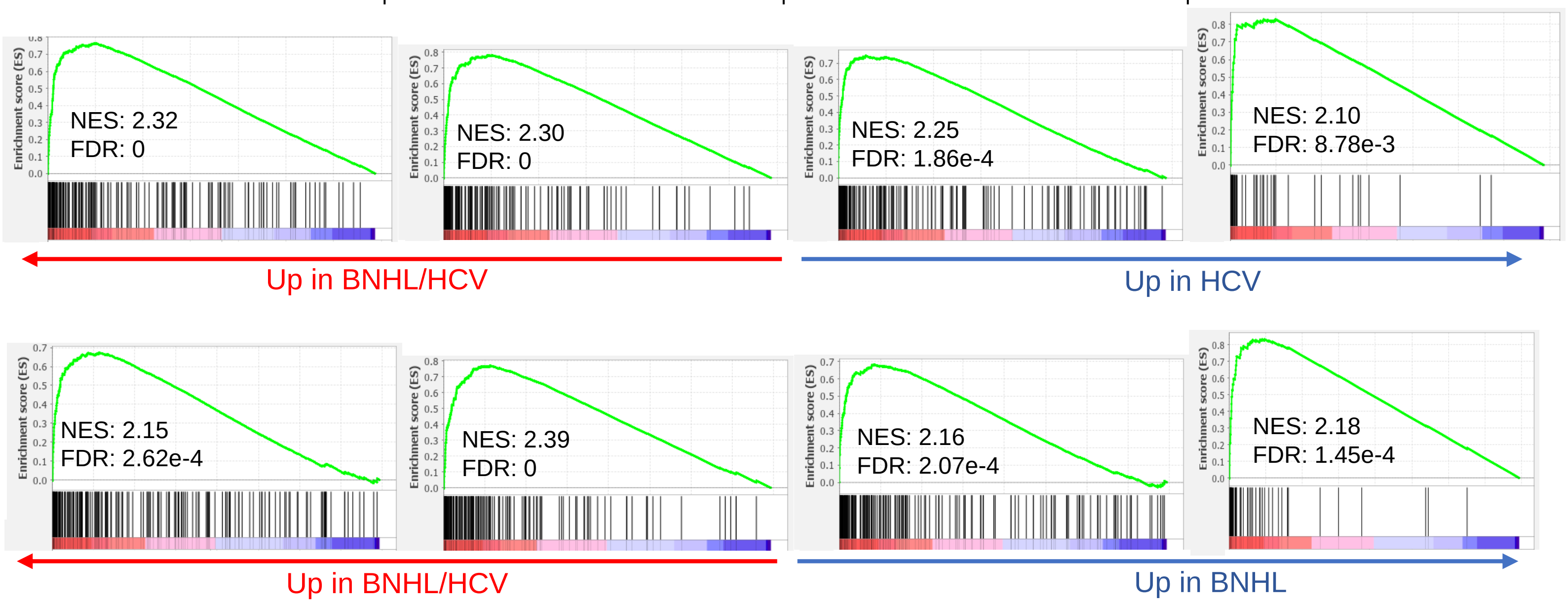

Figure S4 – BNHL/HCV peripheral B cells demonstrate an enhanced proliferative signature. Related to Figure 3. GSEA plots for expressed genes (ranked by fold change) in BNHL/HCV vs HCV (top) and BNHL (bottom) comparisons. Significant gene sets shown come from the GSEA Molecular Signatures Database C2 collection, and all are related to proliferation-associated cancers. Gene set names are listed above the plot, along with a brief description. FDR, false discovery rate; NES, normalized enrichment score

Supplemental References:

1. Bolen, C.R., Robek, M.D., Brodsky, L., Schulz, V., Lim, J.K., Taylor, M.W., and Kleinstein, S.H. (2013). The blood transcriptional signature of chronic hepatitis C virus is consistent with an ongoing interferon-mediated antiviral response. J Interferon Cytokine Res 33, 15-23.
2. Isnardi, I., Ng, Y.S., Menard, L., Meyers, G., Saadoun, D., Srdanovic, I., Samuels, J., Berman, J., Buckner, J.H., Cunningham-Rundles, C., et al. (2010). Complement receptor 2/CD21- human naive B cells contain mostly autoreactive unresponsive clones. Blood 115, 5026-5036.
3. Charles, E.D., Brunetti, C., Marukian, S., Ritola, K.D., Talal, A.H., Marks, K., Jacobson, I.M., Rice, C.M., and Dustin, L.B. (2011). Clonal B cells in patients with hepatitis C virus-associated mixed cryoglobulinemia contain an expanded anergic CD21low B-cell subset. Blood 117, 5425-5437.
